# The Improved Properties of Carboxymethyl Bacterial Cellulose Films with Thickening and Plasticizing

**DOI:** 10.3390/polym14163286

**Published:** 2022-08-12

**Authors:** Zhenbing Sun, Zhengjie Tang, Xiaoping Li, Xiaobao Li, Jeffrey J. Morrell, Johnny Beaugrand, Yao Yao, Qingzhuang Zheng

**Affiliations:** 1 Yunnan Key Laboratory of Wood Adhesives and Glue Products, Southwest Forestry University, Kunming 650224, China; 2 International Joint Research Center for Biomass Materials, Southwest Forestry University, Kunming 650224, China; 3 National Centre for Timber Durability and Design Life, University of the Sunshine Coast, Brisbane, QLD 4102, Australia; 4 Biopolymères Interactions Assemblages (BIA), INRA, Rue de la Géraudière, F-44316 Nantes, France

**Keywords:** glycerin, sodium alginate, tensile strength, elongation at break

## Abstract

This study aims to improve the thermal stability and mechanical properties of carboxymethyl bacterial cellulose (CMBC) composite films. Experiments were conducted by preparing bacterial cellulose (BC) into CMBC, then parametrically mixing sodium alginate/starch/xanthan gum/gelatin and glycerin/sorbitol/PEG 400/PEG 6000 with CMBC to form the film. Scanning electron microscopy, X-ray diffractometry, infrared spectroscopy, mechanical tests, and thermogravimetric analysis showed that the composite films had better mechanical properties and thermal stability with the addition of 1.5% CMBC (% *v*/*v*), 1% sodium alginate, and 0.4% glycerin. Tensile strength was 38.13 MPa, the elongation at break was 13.4%, the kinematic viscosity of the film solution was 257.3 mm^2^/s, the opacity was 4.76 A/mm, the water vapor permeability was 11.85%, and the pyrolysis residue was 45%. The potential causes for the differences in the performance of the composite films were discussed and compared, leading to the conclusion that CMBC/Sodium alginate (SA)/glycerin (GL) had the best thermal stability and mechanical properties.

## 1. Introduction

Bacterial cellulose (BC) is a porous reticulated nanoscale biopolymer synthesized by microbial fermentation [1]. It is a straight-chain molecule consisting of β-D-glucose bound by β-1,4-glycosidic bonds and is also known as β-1,4-glucan [2]. It was first discovered by the British scientist A.J. Brown in 1886 and was identified by physical and chemical analysis as a hydrolyzed cellulose-like substance, with glucose as the main component of the hydrolysate [3]. Bacterial cellulose consists of unique filamentous fibers with a diameter of 0.01–0.10 μm, two to three orders of magnitude smaller than vegetable cellulose. Each filamentous fiber consists of several microfibers in a mesh structure [4]. In addition, bacterial cellulose has high crystallinity, good elasticity and mechanical properties [5], biodegradability, low cost and toxicity [6], and high biocompatibility [7], making it useful as a medical packaging material.

Carboxymethyl cellulose (CMC) is an industrially essential biopolymer resulting from the partial substitution of the 2, 3, and 6 hydroxyl groups on the glucose unit of cellulose by the carboxymethyl group [8,9]. To reduce the use of plastics, researchers have studied a wide range of materials such as CMC, gelatin, carrageenan, starch films, and other biosourced polymers. However, all pure polymer films have some drawbacks, such as the brittleness of gelatin [10,11], the poor thermal stability of carrageenan films [12,13], and the poor water solubility and weak mechanical properties of CMC and starch films [14,15,16,17]. The tensile strength of pure CMC films is 6–9 MPa [18,19,20,21], which cannot meet the requirements for most commercial applications. Modification of CMC-based composite films has been explored by adding different materials to produce composite films with improved mechanical properties [22], thermal stability [23], and degradability, potentially making these materials useful for food preservation [24].

Many studies have been carried out on CMC-based composite films. Rungsiri et al. prepared CMC from durian peel and combined it with rice starch to produce composite films [25]. The performance of these composite films varied between starch and CMC blends, as the amounts of straight-chain and branched-chain polymers in the starch affected functional properties and interactions with other materials [26]. The composite film was tested on tomatoes for its ability to inhibit spoilage and quality loss. Nazmi et al. showed that the addition of gelatine to CMC reduced the flexibility of the film but improved the tensile strength, puncture resistance, and thermal stability [27]. In addition, different gelatin sources had different effects on the mechanical properties of the films. In summary, adding starch, gelatine, and sodium alginate to most CMC films improves their mechanical properties and thermal stability [28,29]. However, BC is also a type of cellulose. There are few reports on the preparation of CMBC and its compounding with macromolecular polymers to produce films with improved mechanical properties and thermal stability. 

## 2. Materials and Methods 

### 2.1. BC Preparation

**Figure 1:**
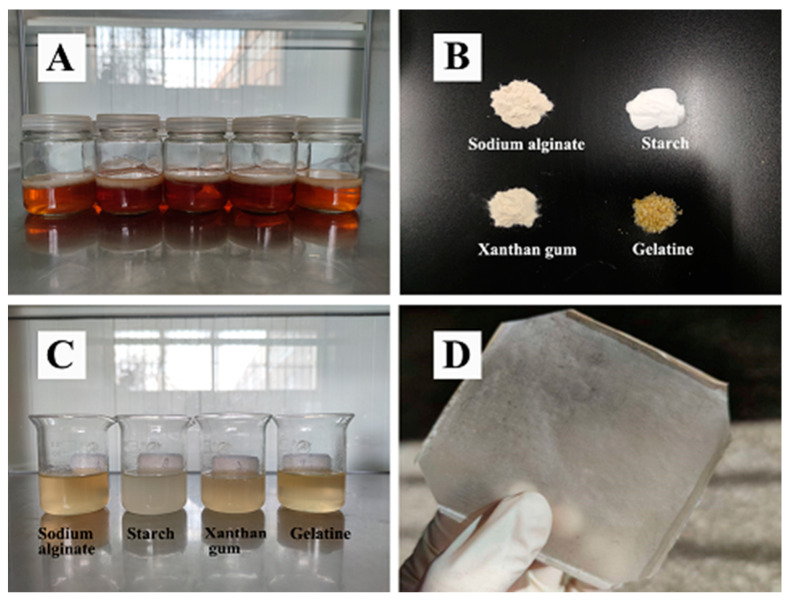
(**A**) Examples of BC (Contains water), (**B**) Sodium alginate, starch, xanthan gum, and gelatin, (**C**) Composite film solution, (**D**) CMBC/SA/GL composite film.

BC made from *Taonella mepensis* was supplied by Beinacruz Biotechnology Co., Ltd. (Suzhou, Jiangsu Province, China). A batch of 54 experimental bottles (cylindrical containers with a diameter of 6cm and a height of 8 cm) was cultivated by autoclaving, inoculating with the bacterium, incubating at 120 °C for 30 min, and finally incubating for 7 days at 37 °C in a medium containing: 20.0 g glucose (Tianjin Fuyu Fine Chemical Co., Ltd., Tianjin, China), 5.0 g yeast paste (Beijing Aoboxing Bio-tech Co., Ltd., Beijing, China), 1.0 g K_2_HPO_4_ (Shanghai Aladdin Biochemical Technology Co., Ltd., Shanghai, China), 15.0 g MgSO_4_ (Tianjin Windship Chemical Reagent Technology Co., Ltd., Tianjin, China), 5 mL anhydrous ethanol (Tianjin Fuyu Fine Chemical Co., Ltd., Tianjin, China), and 1.0 L distilled water at pH 4.5. The wet BC (Figure 1A) was taken out and soaked in distilled water, and the distilled water was changed every hour for 12 consecutive times. The wet BC was cut into 3 cm-diameter pieces that were treated with 1% sodium hydroxide solution (Tianjin Fuyu Fine Chemical Co., Ltd., Tianjin, China) at 80 °C for 1 h. The treated BC was soaked in an excess of distilled water, which was changed every 2 h until the pH was 7. The BC gel was drained and vacuum-dried for 72 h at −80 °C before being crushed into a powder using a high-speed pulverizer (Yongkang Pu’ou Hardware Products Co., Ltd., Jinhua, Zhejiang Province, China). The ground material was passed through a 60-mesh sieve, and prepared for use. 

### 2.2. Carboxymethyl Cellulose BC (CMBC) Preparation

8 g BC, 160 mL of 95% ethanol (Tianjin Fuyu Fine Chemical Co., Ltd., Tianjin, China), and 40 mL of 30% NaOH (Tianjin Fuyu Fine Chemical Co., Ltd., Tianjin, China) solution were mixed and stirred for 60 min at 30 °C. Then 10 g of sodium chloroacetate (Shanghai Macklin Biochemical Co., Ltd., Shanghai, China)were added and the temperature was increased to 65 °C and stirred for 3 h. Glacial acetic acid (90%) (Tianjin Hengxing Chemical Preparation Co., Ltd., Tianjin, China) solution was added to reduce the pH of the mixture and then the samples were washed with ethanol (Tianjin Fuyu Fine Chemical Co., Ltd., Tianjin, China) until the pH was 7. The neutralized samples were oven-dried at 65 °C and stored for later use.

### 2.3. Preparation of CMBC Composite films

1.5 g of CMBC was placed in a heated magnetic mixer (Shanghai Lichenbang Instrument Technology Co., Ltd, DF-101Z, Shanghai, China) for 30 min at 45 °C at a stirring rate of 900–1000 r/min while different proportions of sodium alginate (Beijing Coolaber Technology Co., Ltd., Beijing, China) (Figure 1B), starch (SR) (Chengdu Jinshan Chemical Reagent Co., Ltd., Chengdu, Sichuan Province, China), xanthan gum (XG) (Shanghai Yuanye Biotechnology Co., Ltd., Shanghai, China), gelatin (GEL) (Tianjin Windship Chemical Reagent Technology Co., Ltd., Tianjin, China) were added to produce a final concentration of 1.5 percent solution (0.2%, 0.4%, 0.7%, 1%, 1.3%, all the above proportions are based on the mass of the solvent, the same below) along with different levels of glycerin (Shanghai Yuanye Biotechnology Co., Ltd., Shanghai, China), sorbitol (Tianjin Fuyu Fine Chemical Co., Ltd., Tianjin, China) (SO), PEG 400 (Beijing Coolaber Technology Co., Ltd., Beijing, China), and PEG 6000 (Beijing Coolaber Technology Co., Ltd., Beijing, China) (0.2%, 0.4%, 0.6% ). The mixtures were stirred to dissolve the components and then the film solution (Figure 1C) was placed in an ultrasonic apparatus (Kunshan ultrasonic Intrasonic Co., Ltd., KF-101Z, Kunming, China) operated at 50 HZ for 12 min to remove air bubbles. The film was cast on a PTFE mold (Yangzhong Fuda Insulation Electric Co., Ltd., Yangzhong, Jiangsu Province, China), dried in a blast box (Shanghai Yiheng Scientific Instruments Co., Ltd., Shanghai, China) at 30 °C for 48 h, and then removed (Figure 1D).

### 2.4. Properties of the Composite Films

Tensile strength (MPa) and elongation at break (%) were measured on ten 0.089- to 0.098-mm by 150-mm-long dog-bone samples of each material on a Universal Testing Machine according to procedures described in GB/T 1040.1-20 06 (Plastics Determination of tensile properties). A load was applied to failure at a rate of 1 mm/min. 

The opacity of the CMBC composite films was tested by cutting 10- by 40-mm-long samples and placing them on the inner surface on one side of a cuvette and then measuring absorbance at 600 nm on a 752# ultraviolet spectrophotometer (XP-Spectrum Company, Shanghai, China). Five tests were performed for each material [30]. 

The viscosity of the composite film solution was measured using a Nicolay rotational rheometer meter (MARS60, Thermo Fisher Scientific, Waltham, MA, USA).

Water vapor permeability examined the ability of water vapor to pass through the composite film in order to determine the suitability of each film for maintaining internal moisture.

The water vapor transmission coefficient of the specimen was calculated according to Equation (1) [31].

(1) $$P=\frac{\Delta m\times d}{A\times t\times \Delta P}$$

where P is the water vapor transmission coefficient of the sample in grams of centimeters per square centimeter per second Pascal [g. cm/(cm^2^. s. Pa)],

Δm is the amount of change in the mass of the sample in grams (g) during the period t,

A is the sample area through the water vapor in square meters (m^2^),

t is the difference in time between two intervals after the mass change has stabilized in hours (h),

d is the thickness of the specimen in centimeters (cm), and

ΔP is the difference in water vapor pressure between the two sides of the specimen in Pascal (Pa).

### 2.5. Material Characterization

Microstructure: The composite films were placed on an aluminum grid and examined by field emission scanning electron microscopy on a Nova Nano SEM450 microscope (FEI, Hillsboro, OR, USA). At least five fields were examined for each material.

Fourier Transform Infrared Spectroscopy (FTIR): The composite films were analyzed on a Nicolet i50 FTIR Analyzer (Thermo Scientific, Waltham, MA, USA). The samples were subjected to 64 scans, and the resulting spectra were baseline-corrected and then analyzed for differences in spectra for different raw materials.

X-ray diffraction (XRD): Bacterial cellulose and carboxymethyl bacterial cellulose were examined by X-ray diffractometry on a Rigaku Ultima IV X-ray diffractometer (Rigaku Corp, Tokyo, Japan) (XRD, Ulti,) using a scanning angle from 10° to 40°, a step size of 0.026° (accelerating current = 30 mA and voltage = 40 kV), and Cu-Kα radiation of λ = 0.154 nm.

Thermogravimetric (TG) analysis: Approximately 5.0 to 6.0 mg of the Carboxymethyl BC composite films were ground to pass an 80-mesh to 120-mesh screen and placed into sample holders for analysis on a TGA 92 thermo gravimetric analyzer (KEP Technologies EMEA, Caluire, France). N_2_ was used as the shielding gas and Al_2_O_3_ as the reference compound. The temperature was increased from room temperature (approx. 20–23 °C) to 600 °C at a rate of 20 °C/min to produce thermogravimetric curves. 

## 3. Results and Discussion 

### 3.1. TS and EB of the Composite Films

Tensile strength (TS) and elongation at break (EB) are the basic indicators for evaluating the film properties. Table 1, Table 2, Table 3 and Table 4 show that the additions of glycerin, sorbitol, PEG 400, and PEG 6000 were positively correlated with the elongation at break of the films, and this result is consistent with the results of previous studies [32]. It is worth mentioning that the addition of glycerin had the most significant effect on the elongation at break of the composite films. In addition, the tensile strength of the composite films first increased and then decreased with the addition of sodium alginate, starch, xanthan gum, or gelatin.

**Figure 2:**
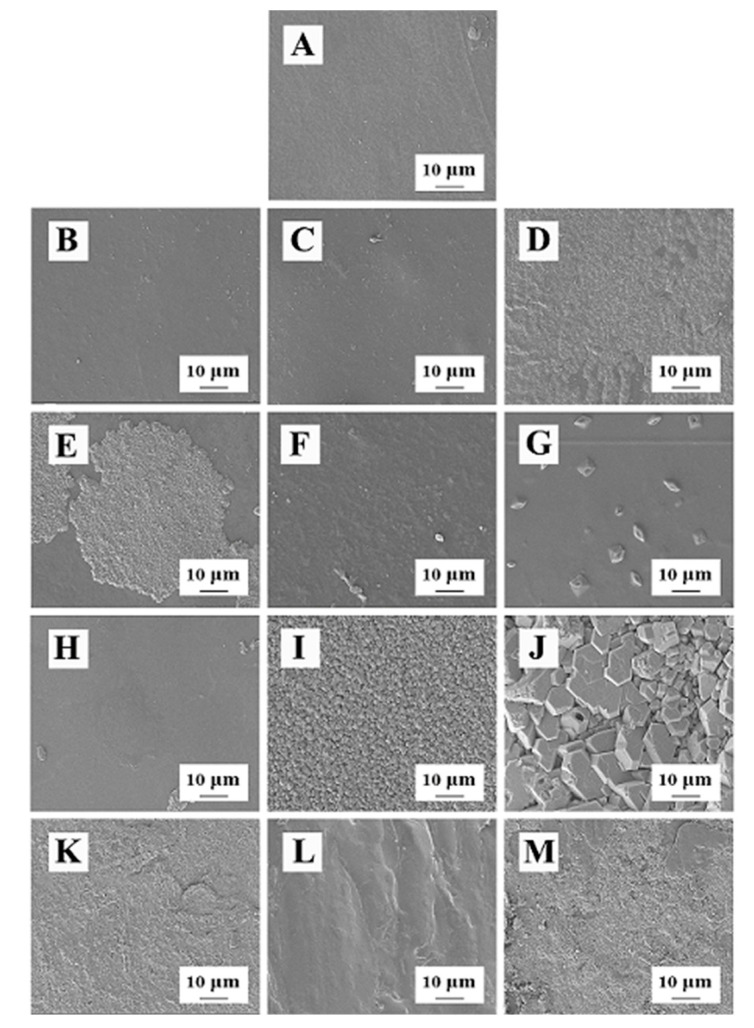
The microstructure of the composite films (**A**): CMBC/SA/Gl; (**B**): CMBC/SR/Gl; (**C**): CMBC/XG/Gl; (**D**): CMBC/GEL/Gl; (**E**): CMBC/SA/SO; (**F**): CMBC/SR/SO; (**G**): CMBC/XG/SO; (**H**): CMBC/GEL/SO; (**I**): CMBC/SA/PEG 400; (**J**): CMBC/SR/PEG 400; (**K**): CMBC/XG/PEG 400; (**L**): CMBC/SA/PEG 6000; (**M**): CMBC/SR/PEG 6000).

Table 5 shows the *p* values and correlation coefficients of the binary regression equation with thickener and plasticizer as independent variables and tensile strength and elongation at break as dependent variables. Thickener and plasticizer were positively correlated with elongation at break of the composite film. The correlation coefficient was above 0.86, and the *p* value was >0.05. In addition, except for CMBC/GEL/GL (Figure 2D), CMBC/SA/PEG 6000 (Figure 2L), and CMBC/SR/PEG 6000 (Figure 2M), the addition of thickeners and plasticizers was positively correlated with tensile strength of the films, with a correlation coefficient above 0.72 and *p* value > 0.05.

Table 6 shows that among the various composite films, the CMBC/SA/GL composite film had the best mechanical properties, with a tensile strength of 38.13 MPa and an elongation at break of 13.40%, at which time sodium alginate (1%) and propanetriol (0.2%) were added. The tensile strength of conventional plastics ranges from 8 MPa to 20 MPa [33]. In comparison, the tensile strength of the composite film was 38.13 MPa, indicating that the composite film had better mechanical properties than conventional plastics.

Table 6 also shows the kinematic viscosity, opacity, and water vapor permeability for each group of best-performing samples. Thickeners had a significant effect on the kinematic viscosity of the film solution for four different thickeners in the film solution in the order of xanthan gum, sodium alginate, starch, and gelatin composite film. The kinematic viscosity of the CMBC/XG/SO composite film was the largest at 1381.95 mm^2^/s, indicating that the solution had poor advective properties. The film did not easily form a smooth surface when poured into the mold and appeared inhomogeneous, typical of non-Newtonian fluids. The kinematic viscosity of the CMBC/SR/PEG 6000 composite film was the smallest, only 27.87 mm^2^/s.

The opacity of each group of composite films is shown in Table 6. The opacity of the laminated film ranged from 4.76 to 11.1 A/mm. Compared with previous studies [34], the opacity of the composite films was higher in all groups because the molecular arrangement of the CMBC was more ordered than that of the CMC. This blocked the passage of visible light [35], making the composite films more crystalline and opaque [36]. In addition, the opacity of the composite films with glycerin as the plasticizer was lower, probably because the glycerin produced composite films with a lower molecular structure that was initially arranged in an orderly manner, thus making it easier for light to pass through.

Crystallinity, molecular size, and other properties of macromolecular polymers all affect the water vapor permeability of the films [37]. The water vapor permeability of the composite films was between 0.04% and 0.12%, lower compared to previous studies [38]. These differences may be due to the more ordered molecular arrangement of CMBC compared to CMC.

### 3.2. Micro-Structure of the Composite Films

Figure 2 shows the composite films prepared with glycerin as the plasticizer, except for the CMBC/GEL/GL composite film, which had many fluid-like attachments on the surface (Figure 2D). The other three composite films had compact surfaces without cracks and bumps (Figure 2A–C), indicating that the components in the composite films were well-integrated and had good interaction forces. In addition, the composite films prepared with sorbitol as the plasticizer formed many round pie-shaped (Figure 2E), crumbled (Figure 2F), granular (Figure 2G), and powder-like substances (Figure 2F,H) on the surface, indicating that some thickeners or CMBC were not incorporated into the composite film system. The composite films prepared with PEG400 and PEG6000 as plasticizers formed a large number of spherical particles (Figure 2I), clay-like materials (Figure 2J), sand-like particles (Figure 2K,M), and ridge-like protrusions (Figure 2L) on the surface, indicating that these composite films had a loose structure and that the components were not sufficiently integrated.

### 3.3. FTIR Spectrum of Composite Films

**Figure 3:**
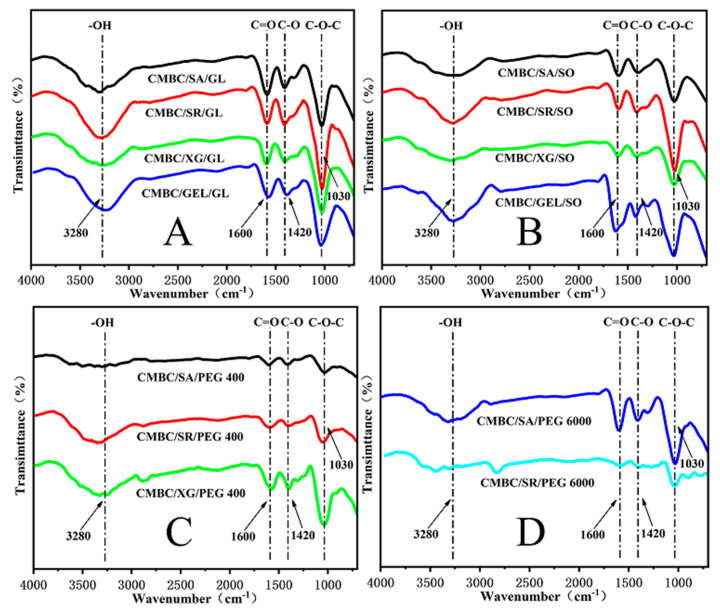
FTIR images of the composite films (**A**): CMBC/SA/G, CMBC/SR/Gl, CMBC/XG/Gl & CMBC/GEL/Gl; (**B**): CMBC/SA/SO, CMBC/SR/SO, CMBC/XG/SO & CMBC/GEL/SO; (**C**): CMBC/SA/PEG 400, CMBC/SR/PEG 400 & CMBC/XG/PEG 400; (**D**): CMBC/SA/PEG 6000 & CMBC/SR/PEG 6000).

The infrared spectra of the composite films are shown in Figure 3. The (O-H) stretching vibration peak of the CMBC composite membrane appears at 3280 cm^−1^, while the (O-H) stretching vibration peak of the carboxymethyl cellulose appears at 3420 cm^−1^ [39], indicating the formation of extensive hydrogen bonds and strong interactions between the additives and the CMC [40]. As can be seen in Figure 3, the addition of sodium alginate/starch/xanthan gum/gelatin and glycerin/sorbitol/PEG 400/PEG 6000 did not change the chemical structure of the CMBC, as can be observed at 1600 cm^−1^ for carboxylate asymmetric stretching and 1420 cm^−1^ and 1310 cm^−1^ for carboxylate asymmetric stretching [41].

### 3.4. XRD-Pattern Characteristics of the Composite Films

**Figure 4:**
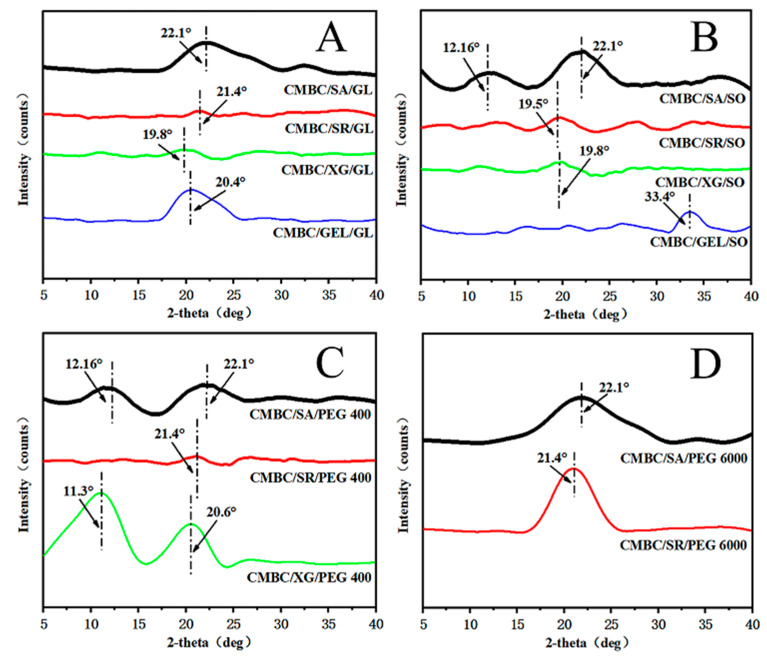
XRD patterns of the composite films (**A**): CMBC/SA/G, CMBC/SR/Gl, CMBC/XG/Gl & CMBC/GEL/Gl; (**B**): CMBC/SA/SO, CMBC/SR/SO, CMBC/XG/SO & CMBC/GEL/SO; (**C**): CMBC/SA/PEG 400, CMBC/SR/PEG 400 & CMBC/XG/PEG 400; (**D**): CMBC/SA/PEG 6000 & CMBC/SR/PEG 6000).

Figure 4 shows the XRD pattern of the composite film. The composite film with glycerol and PEG 6000 as plasticizers showed a broad diffraction peak at diffraction angles of 19.8° to 22.1°, indicating that the components of the composite film were well-bound and strongly interacted with each other (Figure 4A,D; Figure 4B,C). CMBC/CG/PEG 400 showed a broad diffraction peak at 11.3°and 20.6°, respectively [42], both indicating that sodium alginate and xanthan gum were not fully integrated into the composite film system.

### 3.5. The Pyrolysis Characteristics of the Composite Films

**Figure 5:**
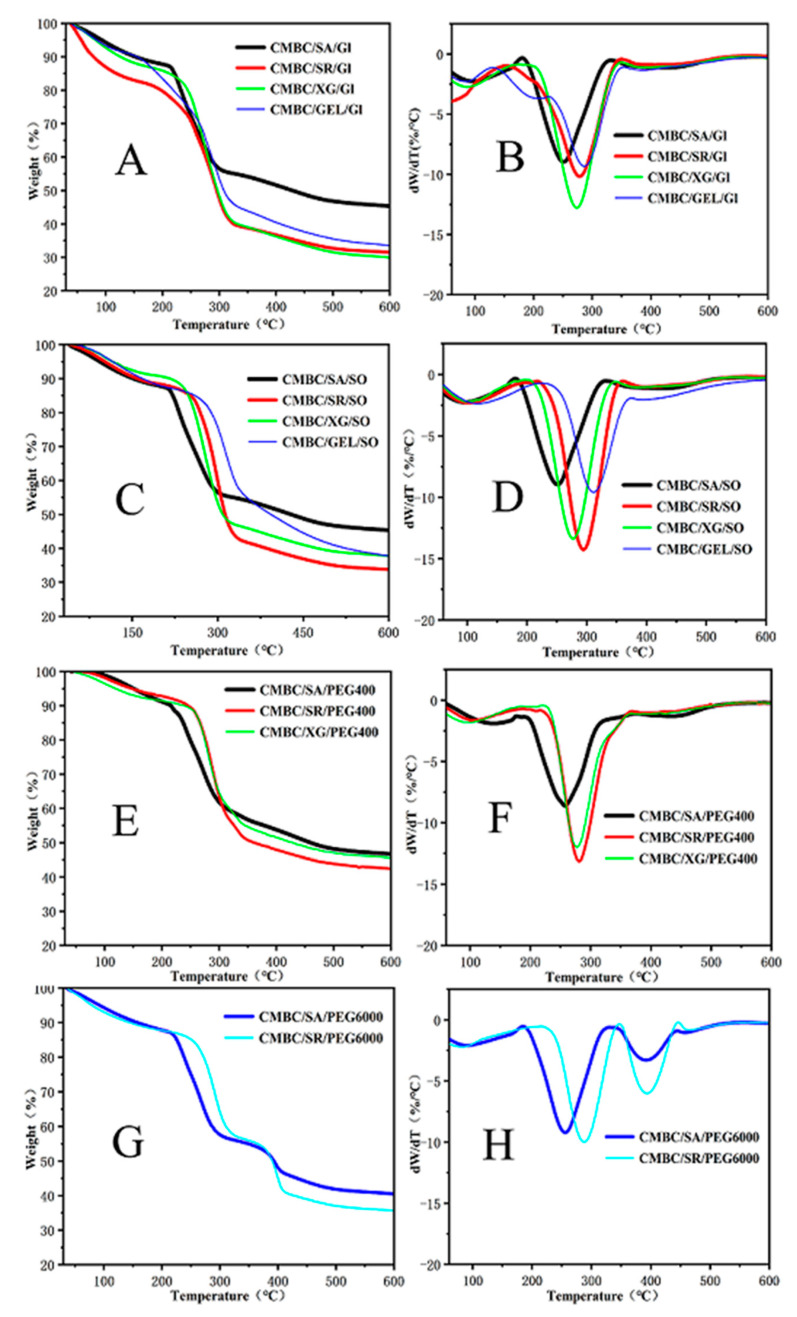
Pyrolysis curves of the composite films ((**A**,**C**,**E**,**G**) for TG; (**B**,**D**,**F**,**H**) for DTG).

Figure 5A–F illustrates the thermal performance of the films. The CMBC/GEL/GL, CMBC/SA/PEG 6000, and CMBC/SR/PEG 6000 composite films all showed two other pyrolysis peaks in addition to a pyrolysis peak of bound water near 100 °C [43], indicating that these three composite films had a nonuniform internal structure leading to poor thermal stability. However, the initiation temperature of the CMBC/SR/GL composite film was significantly lower than the temperatures of the other composite films. The main reason for this is that some of the starch was not dissolved during the preparation of the composite films and collected on the film surface. The TDG-TG diagram shows that the pyrolysis peak of the composite film was between 250 °C and 311 °C. In addition, from the DTG-TG curves in Figure 5 and related calculations, the following main parameters commonly used to reflect the pyrolysis characteristics can be obtained:

the initial temperature of pyrolysis Ts;

the maximum rate of pyrolysis weight loss (d_w_/d_t_)_max_;

the peak temperature Tmax corresponding to (d_w_/d_t_)_max_;

the average rate of pyrolysis weight loss (d_w_/d_t_)_mean_;

the pyrolysis maximum weight loss rate V_∞_;

the temperature interval∆T1/2 corresponding to (d_w_/d_t_)/(d_w_/d_t_)_max_ = 1/2.

The data show that the initial temperature Ts of the pyrolysis of the composite films ranged from 213 °C to 277 °C (Table 6). The maximum weight loss rate V_∞_ of the pyrolysis ranged from 53% to 72%, while the weight loss of the CMBC/SA/PEG400, CMBC/XG/PEG 400, and CMBC/SA/GL composite films was the smallest, at 53%, 54%, and 55%, respectively, indicating that these films had better thermal stability. The pyrolysis temperatures of conventional plastics ranged between 130 °C–145 °C [44]. In contrast, the pyrolysis temperatures of the composite films were 250 °C–300 °C, indicating that composite films have better thermal stability than traditional plastics. The above pyrolysis-related parameters can be combined to develop a comprehensive index D to characterize the degree of difficulty of the pyrolysis of composite films.

(2) $$D=\frac{{\left(d_{w}/d_{t}\right)}_{\max}{\left(d_{w}/d_{t}\right)}_{\mathrm{mean}}V_{\infty }{}_{\;}}{T_{S}T_{\max}\Delta T_{1/2}}$$

Equation (2) shows that the lower the T_S_ (corresponding to a more significant value of D), the easier it is to pyrolyze, (d_w_/d_t_)_ma×_ and (d_w_/d_t_)_mean_, and the more intense the pyrolysis (corresponding to a more considerable D value). The lower values also indicate that the more significant the V_∞_, the more pyrolysis (corresponding to a more considerable D value); the smaller the T_max_ and ∆T_1/2_, the earlier and more concentrated the pyrolysis peaks appear and the more favorable the pyrolysis and gasification. The D values calculated from the above equation were also included in Table 7, which showed that CMBC/SA/PEG 400 and CMBC/SA/GL had the smallest D values of 1.65 × 10^−6^ and 1.92 × 10^−6^, indicating that these two composite films had the best thermal stability.

## 4. Conclusions

A carboxymethyl BC–based composite film was successfully prepared with good thermal stability and mechanical properties. The effects of the addition of sodium alginate/starch/xanthan gum/gelatin and propanetriol/sorbitol/PEG 400/PEG 6000 on the mechanical strength and thermal stability of the carboxymethyl BC–based composite film were investigated. The prepared composite films had suitable mechanical strength and thermal stability when 1.0% sodium alginate and 0.2% propanetriol were added. Regression analysis of the data on mechanical properties yielded a significant correlation between thickeners and plasticizers on the tensile strength and elongation at the break of the composite films (Table 5). The effects of the simultaneous addition of various thickeners on the mechanical properties and thermal stability of composite films will be further studied.

## Figures and Tables

**Table 1:** The effect of GI addition on tensile strength (TS) and elongation at break (EB) of the composite films.

GI Addition	Gl (0.2%)		Gl (0.4%)		Gl (0.6%)	
properties	TS (MPa)	EB (%)	TS (MPa)	EB (%)	TS (MPa)	EB (%)
SA (0.2%)	12.2 (2.28)	29.7 (3.05)	10.6 (2.02)	48.2 (4.09)	6.70 (0.42)	52.5 (4.17)
SA (0.4%)	13.7 (1.19)	25.3 (2.04)	13.6 (2.01)	42.1 (2.89)	13.3 (1.19)	51.4 (3.34)
SA (0.7%)	19.2 (1.23)	23.9 (2.36)	14.9 (2.77)	37.6 (2.49)	12.1 (1.89)	45.6 (3.31)
SA (1.0%)	38.1 (13.4)	25.3 (2.04)	15.1 (1.18)	35.2 (3.35)	15.6 (1.82)	44.3 (3.57)
SA (1.3%)	21.1 (2.05)	10.3 (0.78)	24.6 (2.71)	31.5 (2.55)	15.2 (1.27)	35.4 (2.60)
SR (0.2%)	18.7 (1.71)	33.5 (4.15)	20.8 (3.48)	55.7 (1.68)	12.5 (1.83)	65.7 (9.07)
SR (0.4%)	24.9 (2.13)	25.0 (2.13)	30.1 (5.36)	53.6 (3.57)	20.9 (2.69)	65.0 (3.18)
SR (0.7%)	21.1 (3.01)	16.8 (1.22)	21.4 (3.13)	43.5 (2.24)	10.4 (1.82)	52.6 (2.92)
SR (1.0%)	18.5 (3.13)	10.6 (2.26)	21.2 (2.83)	39.9 (2.56)	10.5 (1.61)	50.0 (1.91)
SR (1.3%)	14.9 (1.26)	7.03 (1.03)	23.8 (1.90)	20.8 (2.81)	8.90 (0.72)	15.8 (1.59)
XG (0.2%)	20.6 (2.67)	6.98 (0.84)	29.7 (2.45)	29.1 (2.74)	10.8 (0.84)	32.0 (2.45)
XG (0.4%)	22.7 (3.65)	7.54 (0.76)	30.3 (5.66)	28.6 (2.38)	12.4 (2.18)	30.8 (2.76)
XG (0.7%)	25.2 (2.79)	8.35 (1.53)	26.6 (2.66)	25.4 (2.49)	14.2 (2.30)	29.3 (2.89)
XG (1.0%)	26.2 (2.93)	9.19 (1.57)	29.4 (4.39)	14.7 (1.43)	13.3 (2.64)	29.9 (2.81)
XG (1.3%)	29.1 (3.93)	6.79 (1.01)	30.1 (3.25)	13.0 (2.64)	16.1 (2.99)	21.5 (3.69)
GEL (0.2%)	15.3 (3.15)	23.9 (4.91)	10.8 (0.89)	25.9 (1.65	11.9 (1.23)	27.0 (2.78)
GEL (0.4%)	30.6 (0.24)	46.2 (5.08)	13.2 (2.28)	49.8 (4.94)	10.7 (1.48)	57.8 (6.03)
GEL (0.7%)	15.6 (1.30)	18.7 (1.09)	19.7 (1.89)	19.0 (2.34)	21.3 (3.26)	35.7 (4.67)
GEL (1.0%)	7.01 (2.05)	10.8 (1.84)	15.1 (1.18)	32.2 (2.65)	15.8 (1.22)	23.6 (2.44)
GEL (1.3%)	10.7 (2.60)	14.6 (2.85)	27.0 (3.85)	38.4 (0.42)	14.1 (3.69)	13.6 (0.42)

Numbers in parentheses are standard deviations.

**Table 2:** The effect of SO addition on the tensile strength (TS) and elongation at break (EB) of the composite films.

SO Addition	SO (0.2%)		SO (0.4%)		SO (0.6%)	
properties	TS (MPa)	EB (%)	TS (MPa)	EB (%)	TS (MPa)	EB (%)
SA (0.2%)	24.6 (3.97)	24.4 (0.75)	24.0 (4.88)	30.5 (2.51)	22.1 (3.77)	45.8 (0.36)
SA (0.4%)	25.6 (4.65)	15.9 (1.90)	18.8 (1.94)	29.7 (2.11)	17.5 (0.27)	32.4 (2.93)
SA (0.7%)	26.2 (0.03)	13.7 (0.37)	24.1 (3.13)	20.5 (1.94)	16.9 (1.26)	29.5 (1.57)
SA (1.0%)	26.9 (2.67)	7.93 (0.89)	26.0 (3.71)	10.7 (1.06)	13.3 (0.99)	23.5 (1.12)
SA (1.3%)	26.4 (3.74)	6.09 (1.57)	16.6 (1.72)	4.24 (0.69)	14.0 (0.83)	16.9 (1.01)
SR (0.2%)	20.8 (1.71)	9.80 (1.58)	24.5 (2.89)	27.6 (2.65)	18.7 (1.85)	32.1 (5.68)
SR (0.4%)	26.6 (4.01)	7.56 (1.48)	27.0 (2.91)	21.1 (2.39)	26.7 (3.47)	26.8 (4.74)
SR (0.7%)	25.9 (4.27)	5.98 (0.34)	26.2 (2.30)	16.2 (1.65)	26.1 (1.93)	25.1 (3.90)
SR (1.0%)	21.2 (2.94)	4.35 (0.84)	24.5 (2.73)	9.01 (2.25)	20.8 (2.10)	13.9 (1.87)
SR (1.3%)	26.6 (3.00)	4.21 (1.67)	26.6 (2.19)	4.29 (0.60)	19.1 (2.14)	6.67 (0.84)
XG (0.2%)	18.6 (3.56)	7.09 (0.95)	20.8 (1.32)	8.08 (1.08)	16.9 (1.63)	8.78 (0.96)
XG (0.4%)	19.9 (0.92)	3.02 (0.54)	25.3 (2.05)	7.01 (1.38)	20.5 (2.08)	7.32 (0.95)
XG (0.7%)	16.5 (3.67)	1.53 (0.34)	20.6 (2.08)	3.00 (0.56)	19.4 (1.44)	4.88 (1.20)
XG (1.0%)	12.3 (2.30)	1.51 (0.29)	21.6 (0.55)	2.80 (0.13)	14.7 (1.24)	3.15 (0.91)
XG (1.3%)	12.8 (1.36)	1.16 (0.10)	19.6 (2.27)	1.83 (0.43)	25.1 (2.31)	2.29 (0.53)
GEL (0.2%)	14.1 (1.50)	13.8 (1.85)	16.0 (0.70)	18.1 (2.73)	7.55 (1.34)	25.1 (3.42)
GEL (0.4%)	9.11 (1.49)	6.45 (1.42)	9.67 (0.78)	8.13 (1.82)	10.7 (2.38)	20.5 (2.72)
GEL (0.7%)	10.5 (1.43)	5.87 (0.45)	18.9 (1.89)	8.67 (1.53)	11.9 (2.87)	9.10 (1.86)
GEL (1.0%)	8.10 (2.45)	5.56 (0.89)	25.8 (1.87)	6.77 (0.07)	16.1 (1.12)	3.62 (0.05)
GEL (1.3%)	7.42 (1.73)	2.75 (0.74)	29.1 (3.29)	4.37 (0.38)	23.6 (2.56)	5.29 (0.62)

Numbers in parentheses are standard deviations.

**Table 3:** The effect of PEG400 addition on the tensile strength (TS) and elongation at break (EB) of the composite films.

PEG 400 Addition	PEG 400 (0.2%)		PEG 400 (0.4%)		PEG 400 (0.6%)	
properties	TS (MPa)	EB (%)	TS (MPa)	EB (%)	TS (MPa)	EB (%)
SA (0.2%)	23.2 (0.99)	6.66 (1.31)	18.2 (3.79)	8.39 (1.66)	14.8 (3.32)	14.7 (3.38)
SA (0.4%)	16.4 (4.12)	3.67 (0.32)	18.0 (3.25)	5.14 (0.51)	11.2 (1.41)	10.7 (5.09)
SA (0.7%)	24.1 (1.30)	6.54 (0.95)	21.4 (3.45)	1.43 (0.42)	19.5 (1.53)	9.23 (1.28)
SA (1.0%)	28.0 (1.68)	3.09 (0.66)	26.5 (4.19)	5.42 (0.66)	17.9 (0.90)	6.22 (1.21)
SA (1.3%)	26.9 (1.18)	1.40 (0.58)	26.1 (1.67)	2.13 (0.34)	20.4 (3.77)	3.77 (0.39)
SR (0.2%)	17.9 (3.34)	13.4 (2.66)	18.4 (1.23)	10.0 (0.60)	15.1 (1.65)	3.94 (0.96)
SR (0.4%)	19.6 (0.66)	8.70 (0.46)	19.9 (0.93)	7.30 (1.05)	18.0 (2.05)	6.64 (0.54)
SR (0.7%)	17.6 (0.48)	2.50 (0.21)	17.0 (0.63)	2.50 (0.21)	17.1 (0.64)	2.33 (0.07)
SR (1.0%)	19.3 (0.65)	2.19 (0.70)	15.8 (1.21)	1.57 (0.23)	14.3 (2.89)	1.35 (0.29)
SR (1.3%)	20.1 (2.45)	1.06 (0.53)	18.04 (2.01)	1.50 (0.34)	19.3 (1.56)	1.21 (0.72)
XG (0.2%)	10.9 (0.53)	2.92 (0.20)	13.9 (0.54)	2.48 (1.08)	9.64 (1.31)	2.98 (0.11)
XG (0.4%)	8.04 (0.64)	1.15 (0.06)	17.8 (0.14)	1.39 (0.23)	16.6 (1.34)	1.50 (0.19)
XG (0.7%)	9.33 (0.81)	0.60 (0.04)	16.7 (1.05)	0.75 (0.06)	14.7 (0.19)	1.09 (0.31)
XG (1.0%)	9.52 (0.15)	0.51 (0.07)	10.7 (1.87)	0.54 (0.02)	8.76 (0.89)	0.98 (0.06)
XG (1.3%)	9.75 (0.75)	0.73 (0.21)	10.4 (1.14)	0.72 (0.54)	12.6 (1.32)	0.74 (0.24)
GEL (0.2%)	—	—	—	—	—	—
GEL (0.4%)	—	—	—	—	—	—
GEL (0.7%)	—	—	—	—	—	—
GEL (1.0%)	—	—	—	—	—	—
GEL (1.3%)	—	—	—	—	—	—

Numbers in parentheses are standard deviations. —: Indicates that some samples in this group did not form a composite film.

**Table 4:** The effect of PEG6000 addition on the tensile strength (TS) and elongation at break (EB) of the composite films.

PEG 6000 Addition	PEG 6000 (0.2%)		PEG 6000 (0.4%)		PEG 6000 (0.6%)	
properties	TS (MPa)	EB (%)	TS (MPa)	EB (%)	TS (MPa)	EB (%)
SA (0.2%)	13.6 (0.68)	9.74 (0.65)	31.8 (2.13)	3.59 (0.29)	23.9 (0.63)	1.77 (0.17)
SA (0.4%)	14.7 (0.83)	4.77 (0.36)	16.7 (1.56)	2.14 (0.54)	15.9 (2.08)	2.07 (0.20)
SA (0.7%)	23.4 (4.17)	2.53 (0.61)	16.5 (0.95)	2.34 (0.02)	18.4 (0.89)	2.33 (0.25)
SA (1.0%)	25.5 (1.10)	3.20 (0.30)	17.8 (0.93)	2.05 (0.05)	13.2 (0.41)	1.14 (0.19)
SA (1.3%)	20.5 (0.29)	2.07 (0.18)	18.8 (1.32)	2.04 (0.01)	10.9 (0.68)	0.75 (0.16)
SR (0.2%)	21.9 (1.67)	2.22 (0.25)	28.8 (1.17)	3.46 (0.38)	29.0 (1.93)	4.99 (0.69)
SR (0.4%)	25.6 (1.14)	2.11 (0.50)	30.1 (1.20)	2.97 (0.27)	21.4 (0.76)	3.36 (0.76)
SR (0.7%)	28.1 (2.05)	2.50 (0.37)	28.9 (0.61)	3.02 (0.55)	17.3 (1.90)	3.06 (0.38)
SR (1.0%)	26.2 (1.41)	1.38 (0.15)	22.1 (0.88)	2.04 (0.30)	10.6 (1.43)	2.11 (0.20)
SR (1.3%)	25.2 (0.51)	1.41 (0.20)	21.4 (2.10)	1.04 (0.34)	20.1 (2.34)	1.52 (0.23)

Numbers in parentheses are standard deviations.

**Table 5:** *p* value and Correlation coefficients for the effects of thickeners and plasticizers on tensile strength (TS) and elongation at break (EB) of composite films.

Composite Film	TS (MPa)		EB (%)	
	*p* Value	Correlation Coefficient	*p* Value	Correlation Coefficient
CMBC/SA/Gl	<0.0001	0.94	<0.0001	0.99
CMBC/SR/Gl	0.0019	0.95	<0.0001	0.99
CMBC/XG/Gl	0.0002	0.97	0.0001	0.98
CMBC/GEL/Gl	>0.05	—	0	0
CMBC/SA/SO	0.0008	0.91	<0.0001	0.99
CMBC/SR/SO	0.0164	0.90	<0.0001	0.98
CMBC/XG/SO	<0.0001	0.99	0.0002	0.98
CMBC/GEL/SO	0.0004	0.97	<0.0001	0.99
CMBC/SA/PEG400	<0.0001	0.93	0.0022	0.95
CMBC/SR/PEG400	0.0251	0.72	0.0003	0.97
CMBC/XG/PEG400	0.0114	0.91	<0.0001	0.99
CMBC//GEL/PEG400	—	—	—	—
CMBC/SA/PEG6000	>0.05	—	0.0057	0.86
CMBC/SR/PEG6000	>0.05	—	<0.0001	0.96

—: Indicates that when *p* value > 0.05, there is no correlation coefficient.

**Table 6:** The physical properties of CMBC composite films with difference additives.

Composite Film	Amount of Plasticizer Added (%)	Amount of Thickener Added (%)	The Kinematic Viscosity (mm^2^/s)	Opacity (A/mm)	Water Vapor Permeability g·cm/(cm^2^·s·Pa)
CMBC/SA/Gl	1	0.2	257.3 (3.34)	4.76 (1.11)	0.12 (0.02)
CMBC/SR/Gl	0.4	0.4	52.9 (5.38)	5.00 (0.38)	0.07 (0.01)
CMBC/XG/Gl	0.4	0.4	1018.1 (19.4)	5.93 (0.57)	0.11 (0.03)
CMBC/GEL/Gl	0.4	0.2	39.1 (3.82)	5.21 (0.31)	0.08 (0.02)
CMBC/SA/SO	1	0.2	821.0 (8.53)	5.76 (0.45)	0.10 (0.07)
CMBC/SR/SO	0.4	0.4	43.9 (2.32)	11.1 (0.53)	0.06 (0.01)
CMBC/XG/SO	0.4	0.4	1382.0 (21.70)	6.79 (0.14)	0.06 (0.03)
CMBC/GEL/SO	0.3	0.4	54.0 (4.03)	5.37 (0.43)	0.04 (0.01)
CMBC/SA/PEG400	1	0.2	471.1 (2.95)	4.70 (0.08)	0.07 (0.02)
CMBC/SR/PEG400	1.3	0.2	34.6 (5.34)	9.64 (1.03)	0.1 (0.04)
CMBC/XG/PEG400	0.4	0.4	1238.8 (10.60)	10.2 (1.92)	0.08 (0.03)
CMBC//GEL/PEG400	—	—	—	—	—
CMBC/SA/PEG6000	1	0.2	579.9 (4.31)	5.53 (0.32)	0.11 (0.03)
CMBC/SR/PEG6000	0.4	0.4	27.9 (3.33)	10.1 (0.94)	0.07 (0.02)

The numbers in parentheses are standard deviations. —: Indicates that some samples in this group did not form a composite film.

**Table 7:** Pyrolytic properties of the composite films.

Composite Film	(d_w_/d_t_)_ma×_ (%/°C)	(d_w_/d_t_)_mean_ (%/°C)	V_∞_ (%)	T_S_ (°C)	T_max_ (°C)	∆T_1/2_ (°C)	D
CMBC/ SA/GL	−8.956	−1.59	55	213	250.42	76	1.92 × 10^−6^
CMBC/ SR/GL	−10.1714	−1.91	69	220	278.3	95.6	2.80 × 10^−6^
CMBC/ XG/GL	−12.7741	−1.99	70	232	273.9	85.4	3.96 × 10^−6^
CMBC/ SA/SO	−10.126	−1.83	61	225	264.0	69.8	2.68 × 10^−6^
CMBC/ SR/SO	−14.2546	−1.93	66	265	294.4	70.7	4.53 × 10^−6^
CMBC/ XG/SO	−13.3623	−1.81	72	246	276.2	73.1	4.58 × 10^−6^
CMBC/ GEL/SO	−9.5676	−1.88	62	277	310.4	73.6	2.59 × 10^−6^
CMBC/ SA/PEG400	−8.597	−1.54	53	219	256.3	90.1	1.65 × 10^−6^
CMBC/ SR/PEG 400	−13.1144	−1.65	58	251	280.1	74.2	2.28 × 10^−6^
CMBC/ XG/PEG400	−11.981	−1.58	54	252	277.0	73.2	2.09 × 10^−6^

## Data Availability

The data presented in this study are available from the listed authors.
